# Preparation of Carbon-Covered Phosphorus-Modified Alumina with Large Pore Size and Adsorption of Rhodamine B

**DOI:** 10.3390/nano11030799

**Published:** 2021-03-20

**Authors:** Shuaiqi Chen, Xuhui Wang, Weiyi Tong, Jianchuan Sun, Xiangyu Xu, Jiaqing Song, Jianyi Gong, Wei Chen

**Affiliations:** 1 State Key Laboratory of Chemical Resource Engineering, Beijing University of Chemical Technology, Beijing 100029, China; 2015210377@mail.buct.edu.cn (S.C.); 2016400154@mail.buct.edu.cn (X.W.); 2020700037@mail.buct.edu.cn (J.S.); xuxy@mail.buct.edu.cn (X.X.); 2 State Key Laboratory of Green Chemical Engineering and Industrial Catalysis, Shanghai Research Institute of Petrochemical Technology, SINOPEC, Shanghai 201208, China; tongwy.sshy@sinopec.com; 3 Sinopec Catalyst Co., Ltd., Beijing 100029, China; gongjy@sinopec.com; 4 Department of Physics, The University of Texas at Arlington, 502 Yates Street, 108 Science Hall, Arlington, TX 76019, USA; weichen@uta.edu

**Keywords:** modified alumina, carbon-covered phosphorus-modified alumina, pore size, adsorption, Rhodamine B

## Abstract

In this study, phosphorus-modified alumina with large pore size was synthesized through a coprecipitation method. The carbon-covered, phosphorus-modified alumina with large pores was prepared by impregnating with glucose and carbonizing to further improve the adsorption of organic dyes. The morphology and structure of these composites were characterized by various analysis methods, and Rhodamine B (RhB) adsorption was also examined in aqueous media. The results showed that the specific surface area and pore size of the phosphorus-modified alumina sample AP7 (prepared with a P/Al molar ratio of 0.07) reached 496.2 m^2^·g^−1^ and 21.9 nm, while the specific surface area and pore size of the carbon-covered phosphorus-modified alumina sample CAP7–27 (prepared by using AP7 as a carrier for glucose at a glucose/Al molar ratio of 0.27) reached 435.3 m^2^·g^−1^ and 21.2 nm. The adsorption experiment of RhB revealed that CAP7–27 had not only an equilibrium adsorption capacity of 198 mg·g^−1^, but also an adsorption rate of 162.5 mg·g^−1^ in 5 min. These superior adsorption effects can be attributed to the similar pore structures of CAP7–27 with those of alumina and the specific properties with those of carbon materials. Finally, the kinetic properties of these composites were also studied, which were found to be consistent with a pseudo-second-order kinetic model and Langmuir model for isothermal adsorption analysis. This study indicates that the prepared nanomaterials are expected to be promising candidates for efficient adsorption of toxic dyes.

## 1. Introduction

Rhodamine B (RhB) is a cationic dye that has attracted much attention due to its color and toxicity [1,2]. Currently, RhB is mainly used in the fields of paper-making, preparation of various coatings and lacquers, preparation of textiles, production of leather, and industrial dyeing; these industries are among the main sources of water pollution. When dye effluents from textiles are released into the water, the dyes impede the invasion of sunlight into the water environment, adversely compete with the oxygen transfer, and prohibit the re-oxygenation scope of the receiving water. Ultimately, this occurrence results in the shrinkage of biological movement [3]. At present, many treatment technologies for dye wastewater are available, including coagulation-flocculation [4,5], electrochemical oxidation [6,7], photocatalytic degradation [8,9,10], biological degradation [11,12,13], and adsorption. Compared with the aforementioned methods, adsorption treatment is one of the most promising methods for industrial wastewater treatment due to its low cost, high efficiency, good stability, and easy operation [14,15,16,17,18,19,20,21].

Alumina has been extensively applied in adsorbents, catalysts, humidity sensors, and optical materials due to its large surface area and unique pore structure [22]. Typically, alumina is positively charged on its surface in a neutral or alkaline aqueous solution, leading to its poor adsorption efficiency on cationic dyes. Therefore, alumina-based adsorbents are mainly used to remove anions and anion dyes [22,23]. However, cationic dyes also play a key role in organic dye wastewater, such as RhB. For instance, Lin et al. [24] found that the adsorption capacity of RhB on alumina was just only 3.6 mg·g^−1^. Meanwhile, carbon materials have great adsorption capacity and are commonly used for the adsorption of several organic dyes, but also have slow adsorption rates due to their poor microporous structure [25]. 

Consequently, composite materials were prepared to enhance adsorption performance for cationic dyes because such materials have the potential to combine the advantages of each pristine material. For example, the composite material formed by carbon and alumina can have high a specific surface area, large pore size, and other properties of alumina materials, and can also achieve an adsorption capacity equivalent to that of carbon materials [24]. Therefore, carbon-covered, phosphorus-modified alumina was synthesized by glucose impregnation, and achieved high adsorption capacity for the cationic dye, RhB.

In this work, based on the study reported by Song [26] and Lin [24], different acidic aluminum and alkaline aluminum sources were used to synthesize alumina with large pore size. This process was followed by impregnating with glucose and carbonizing in a tube furnace to obtain carbon covered alumina. The morphology, structure, and adsorption properties of RhB were subsequently characterized by well−established analytical and testing methods. Relevant parameters, including adsorbent dose, contact time, and initial RhB concentrations, were optimized for the best adsorption performance of the composite. The adsorption capacity of CAP is comparable with that of compounds reported in the literature, as shown in Table 1. Furthermore, the kinetics and adsorption isotherms were also investigated.

## 2. Materials and Methods 

### 2.1. Materials

Sodium sulfate (Na_2_SO_4_), aluminum hydroxide (Al(OH)_3_), sodium hydroxide (NaOH), aluminum chloride hexahydrate (AlCl_3_·6H_2_O), disodium hydrogen phosphate dodecahydrate (Na_2_HPO_4_·12H_2_O), and glucose (C_6_H_12_O_6_) were purchased from Xilong chemical company (Shantou, GD, China). All of the materials were of analytical grade.

### 2.2. Synthesis of Phosphorus-Modified Alumina

First, three solutions were separately prepared. The preparation of sodium aluminate solution was as follows: 16.26 g of NaOH, 22.02 g of Al(OH)_3_, and 20.80 g of deionized water were put into a Teflon-lined stainless autoclave and stirred at a speed of 30 r·min^−1^ for 2 h at 160 °C. The preparation of aluminum chloride solution was as follows: 27.77 g of AlCl_3_·6H_2_O was dissolved in 300.0 g of deionized water, and the mixture was stirred until it formed a clear solution. The preparation of disodium hydrogen phosphate solution was as follows: 21.30 g of Na_2_HPO_4_·12H_2_O was dissolved in 200.0 g of deionized water [32].

Aluminum chloride solution was added dropwise to the sodium aluminate solution at room temperature and stirred to achieve the desired pH (8.0–10.0). The suspension was transferred into a Teflon-lined stainless autoclave and then heated at 90 °C for 20 h. The mixture was filtered, washed with water and ethanol, and then dried at 80 °C to obtain boehmite. Different solution conditions were used to study the effects of different phosphorus contents brought by the disodium hydrogen phosphate solution, and the boehmite samples were named P0, P1, P3, P5, P7, P9, and P11 based on different P/Al molar ratios (0, 0.01, 0.03, etc.). Further, the boehmite samples were calcined at 550 °C for 2 h to obtain phosphorus-modified alumina, and the phosphorus-modified alumina samples were noted as AP0, AP1, AP3, AP5, AP7, and AP9 according to P0, P1, P3, P5, P7, and P9, respectively.

### 2.3. Synthesis of Carbon-Covered Phosphorus-Modified Alumina

Boehmite samples were mixed with glucose aqueous. The obtained mixture was dried at 80 °C for 12 h and calcined in a tube furnace at 550 °C for 2 h under N_2_ flow (20 mL·min^−1^). The molar ratio of Al and glucose was varied to study the effects of different glucose contents. The carbon-covered phosphorus-modified alumina synthesized from AP7 were noted as CAP7–23, CAP7–25, CAP7–27, CAP7–30, and CAP7–32 according to different molar ratios of Glu/Al (0.23, 0.25, 0.27, etc.).

### 2.4. Material Characterization

X-ray diffraction analysis was performed using XRD-6100AS (SHIMADZU, Kyoto, Japan) with a Cu anode operating at 40 kV and 40 mA. Data were collected for 2θ values ranging from 5° to 80°, and the goniometer velocity was 5°·min^−1^. Specific surface areas and pore sizes of the samples were measured on a Micromeritics apparatus, the TriStar II 3020 (MICROMERITICS, Norcross, GA, USA), by nitrogen adsorption at 77 k. The samples were degassed under vacuum for 8 h at 300 °C, and the specific surface areas were obtained according to the Brunauer-Emmett-Teller (BET) method. The microstructure was analyzed using a high-resolution transmission electron microscopy JEM-2100 (JEOL, Mitaka, Japan). Zeta potential results were obtained using a ZEN3600 (MAIVERN, Malvern, UK). The sample was dispersed into 0.01 M KNO_3_ solution; the isoelectric point (IEP) was the pH when the zeta potential of the sample was zero, by adjusting with 0.01 M HNO_3_ solution or 0.01 M KOH solution.

### 2.5. Adsorption of RhB

To identify the best adsorption capacity of phosphorus-modified alumina (AP), 20 mg of AP1, AP3, AP5, AP7, and AP9 were separately added to the RhB solution with an initial concentration of 200 mg·L^−1^ for 24 h. Furthermore, the best adsorption capacity of carbon-covered phosphorus-modified alumina (CAP) was determined with the same experimental conditions. Exploring the initial pH effect for adsorption capacity is usually required, because the solution pH influences both surface binding sites of the adsorbent and aqueous chemistry. Herein, the effect of pH on RhB removal was researched by adjusting the initial pH from 3.5 to 9.5 with 0.01 M HCl or 0.01 M NaOH.

To determine the adsorption kinetics of RhB, 20 mg adsorbent was added to 100 mL RhB solution with a concentration of 200 mg∙L^−1^ for various adsorption times, ranging from 5 min to 1140 min. All adsorption experiments were carried out in beakers at 308 K on a multipoint magnetic stirrer with a shaking speed of 300 r∙min^−1^.

After adsorption, the solution with residual RhB was collected by filtration and then evaluated using an ultraviolet spectrophotometer (UV-2600, SHIMADZU, Kyoto, HI, Japan) at 554 nm. 

The adsorption capacity of RhB q_e_ (mg·g^−1^) at equilibrium was calculated by the following Equation (1):

(1) $$q_{e}=\frac{\left(C_{0}-C_{e}\right)\times V}{m}\;$$

The adsorption capacity of RhB q_t_ (mg·g^−1^) at time (t) was calculated by the following Equation (2):

(2) $$q_{t}=\frac{\left(C_{0}-C_{t}\right)\times V}{m},$$

The removal efficiency of RhB was determined by the following Equation (3):

(3) $$R=\frac{C_{0}-C_{t}}{C_{0}}\times 100.$$

where C_0_ (mg·L^−1^) is the initial concentration of RhB, C_e_ (mg·L^−1^) is the equilibrium concentration of RhB, C_t_ is the concentration of RhB at time t (min), V(L) is the total volume of the solution, and m (mg) is the mass of adsorbent.

## 3. Results

### 3.1. Characterizations of Carbon-Covered Phosphorus-Modified Alumina

**Figure 1:**
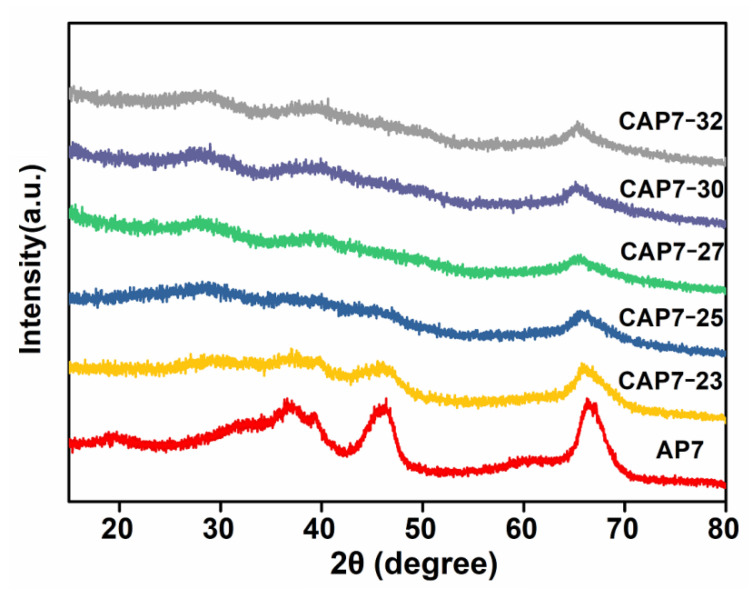
XRD patterns of phosphorus-modified alumina and carbon-covered phosphorus-modified alumina.

Figure 1 depicts the X-ray diffractograms (XRD) of AP7 and CAP7–27, respectively. The XRD pattern of AP7 does not show obvious diffraction peaks assignable to the alumina crystalline phases, implying that the alumina is of low crystallinity. Two broad peaks at approximately 45° and 67°, respectively, are indicated by the pattern and are characteristic peaks of the γ-alumina phase [33,34]. The XRD pattern of CAP7–27 is similar to that of AP7, besides a broad diffraction peak at approximately 25°, which is consistent with the (002) reflection of the graphite lattice [23].

Nitrogen adsorption-desorption isotherms were used to investigate the pore texture of the samples. Table 2 shows a summary of the phosphorus-modified alumina. As shown in Table 2, AP7 had the largest, most probable aperture among these samples. The most probable aperture of AP7 increased to 21.9 nm when the P/Al molar ratio increased from 0 to 0.07. The latter adsorption results suggest that AP7 also had the best adsorption efficiency among these samples. 

**Figure 2:**
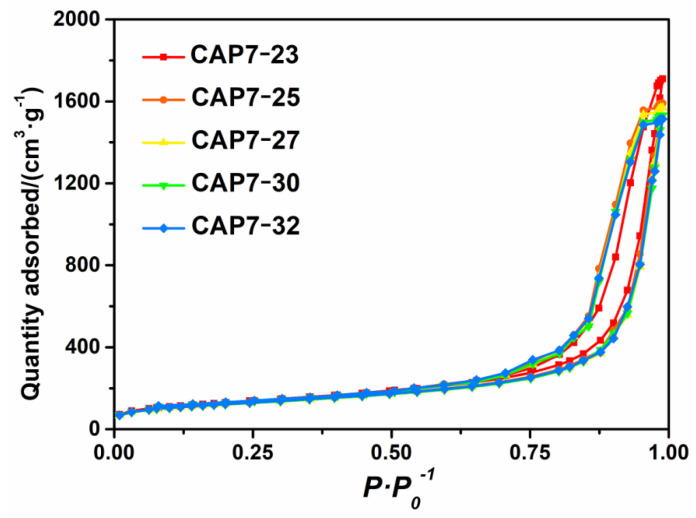
N_2_ adsorption-desorption isotherms of carbon-covered phosphorus-modified alumina.

**Figure 3:**
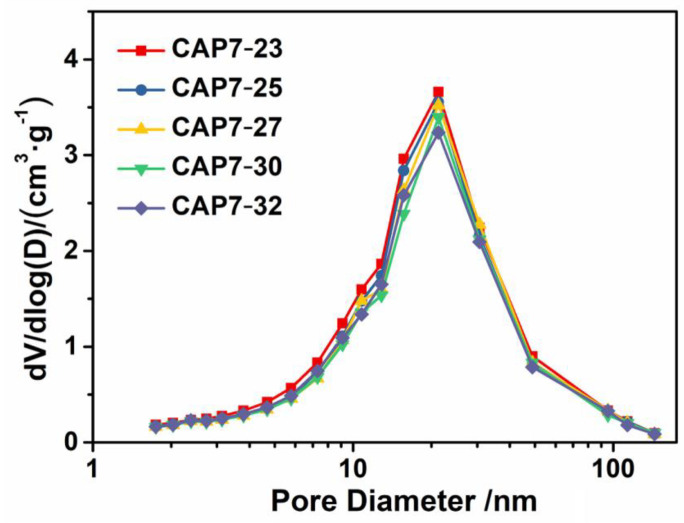
Pore size distributions of carbon-covered phosphorus-modified alumina.

Nitrogen adsorption-desorption isotherms of samples are shown in Figure 2, and both graphs display type IV isotherms. The results showed that all of the samples exhibited isotherms with obvious hysteresis loops. When the glucose impregnation amount was lower than its monolayer dispersion threshold, a uniform thin layer of carbon was formed to cover the alumina surface [35]. However, when the impregnation amount was higher than the monolayer dispersion threshold, it formed not only a uniform carbon thin layer, but also carbon particles which further accumulated to form small pore structures. Figure 3 and Table 3 indicate that the most probable aperture of CAP7 was 21.0 nm. There were no new distribution peaks, indicating that the amount of glucose did not exceed the monolayer dispersion threshold. 

**Figure 4:**
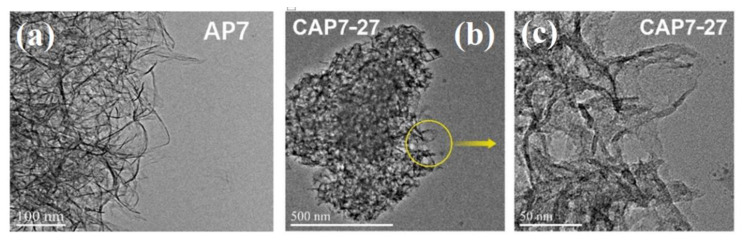
HRTEM image of (**a**) AP7, (**b**) CAP7–27, (**c**) high resolution image of CAP7–27.

Figure 4 shows the HRTEM images of the sample AP7 and CAP7–27, respectively. It can be seen in Figure 4 that the morphology of AP7 was a pleated-sheet structure. Carbon particles could not be seen in the HRTEM images of CAP7–27, and the pore volume of CAP7–27 decreased from 3.03 cm^3^·g^−1^ of AP7 to 2.43 cm^3^·g^−1^, suggesting that the carbon was uniformly dispersed on independent sheets that were stacked on top of each other to form a thicker structure. 

**Figure 5:**
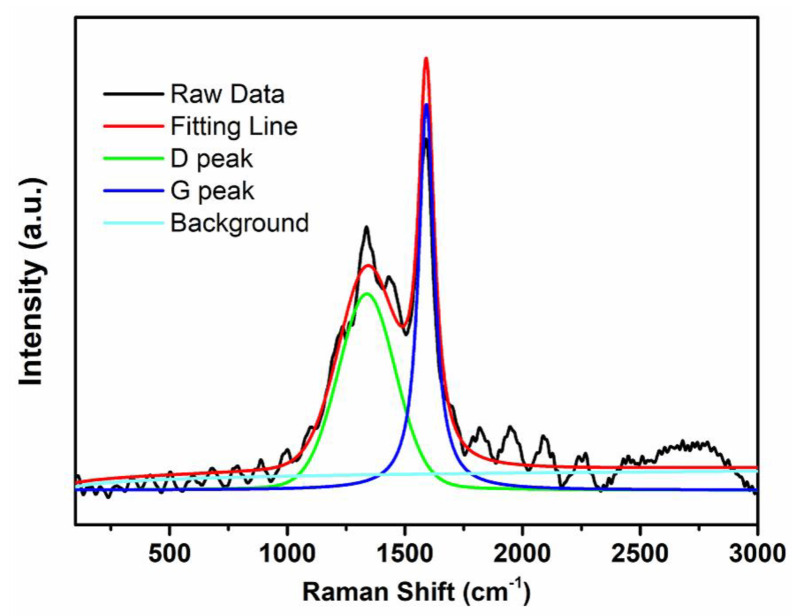
Raman spectrum of CAP7–27.

In the Raman spectrum, the G peak is characteristic of single-crystal graphite at around 1575 cm^−1^. With the increase of graphite lattice defects, edge disordered arrangement, and low symmetry carbon structure, the D peak will appear near 1360 cm^−1^, which represents the disorder degree of carbon material structure. As can be seen in Figure 5, an obvious G peak was observed on the CAP7–27 at 1575 cm^−1^, indicating that the glucose on the sample surface had been carbonized into graphite.

### 3.2. Characterizations of Carbon-Covered Phosphorus-Modified Alumina

**Figure 6:**
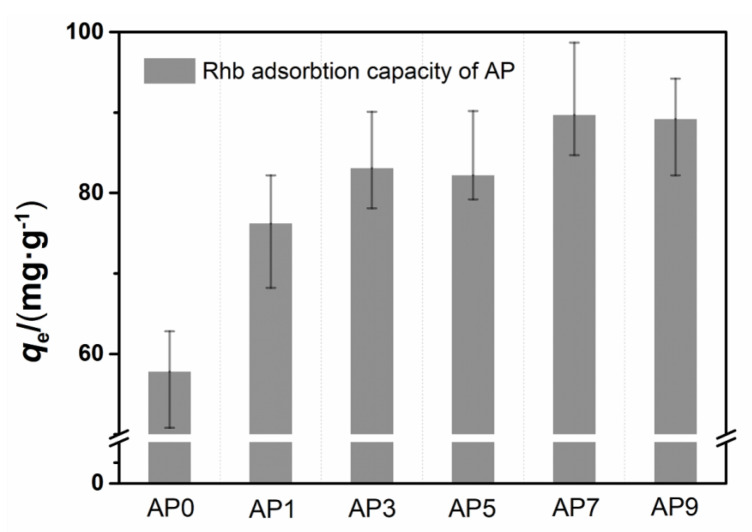
RhB removal efficiency of different phosphorus-modified alumina.

As shown in Figure 6, AP7 had the maximum adsorption capacity of RhB (89.7 mg·g^−1^). Decreasing the isoelectric point could reduce the effect of electrostatic repulsion, thereby increasing the adsorption capacity of RhB.

**Figure 7:**
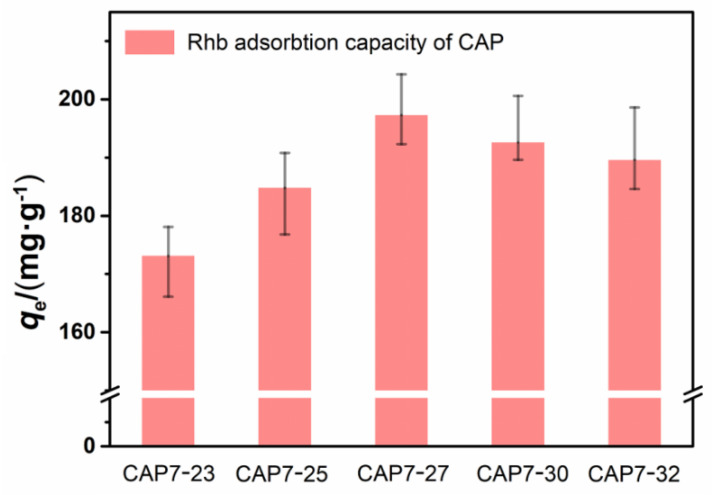
RhB removal efficiency of different carbon-covered phosphorus-modified alumina.

As seen in Figure 7, all the samples had higher adsorption capacities of RhB than AP7 under the same condition as the CAP7–27 sample, reaching the highest equilibrium adsorption capacity of 197.3 mg·g^−1^. The molecular size of RhB is approximately 1.5 nm × 1.2 nm × 0.5 nm, and the dimers of RhB are larger [36]. Because RhB molecules have a large size, the adsorption of RhB was suppressed by the microporous nature of the adsorbent.

Various adsorbents of RhB reported in previous studies were compared, as shown in Table 1. It is indicated that the samples of AP7 and CAP7–27 have great potential, with high adsorption capacity. Both AP7 and CAP7–27 have higher adsorption capacity than the reported alumina adsorbents and modified alumina adsorbents. Compared with other carbon materials and graphene materials, they still held an advantage in adsorption capacity and low cost. Compared with magnesium silicate/carbon composite [27], CAP7–27 had a faster adsorption rate in the first 5 min due to its larger pore size. Compared with Gg-cl-P (AA-co-AAm)/Fe_3_O_4_ nanocomposite [37], both AP7 and CAP7–27 have a simpler preparation process and lower cost.

### 3.3. Adsorption Kinetic Studies

**Figure 8:**
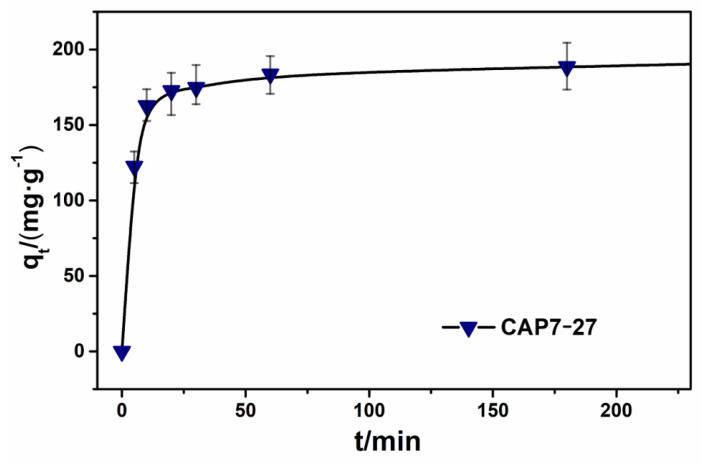
RhB removal capability of CAP7–27 with different contact time.

Figure 8 shows the RhB removal capability of CAP7–27 with different contact times at a temperature of 303 K and an initial RhB concentration of 200 mg·L^−1^. The adsorption capacity was 162.5 mg·g^−1^ at 5 min, which was 82.4% of the equilibrium adsorption capacity of 197.3 mg·g^−1^. The adsorption capacity of RhB increased rapidly between 0−30 min, reaching 174.8 mg·g^−1^ at 30 min, which was 88.6% of the equilibrium adsorption capacity. At 360 min, the adsorption rate was equal to the desorption rate, reaching the equilibrium state. The initial rapid adsorption was due to the existence of large pores. When a sample has a large pore size, RhB can quickly reach active sites at the beginning of adsorption, resulting in a faster adsorption rate [38,39,40,41]. Our results reported here, in addition to the literature [42,43,44,45], prove that porous materials have many applications, from antipollution to solid-state lighting and electronics.

A pseudo-first-order equation is usually defined as follows:

(4) $$\ln\left(q_{e}-q_{t}\right)={\mathrm{lnq}}_{e}-k_{1}t,$$

A pseudo-second-order equation is usually defined as follows:

(5) $$\frac{{\mathrm{dq}}_{e}}{\mathrm{dt}}=k_{2}{\left(q_{e}-q_{t}\right)}^{2},$$

After integration and variation by using boundary conditions of t = 0 min, q_t_ = 0 mg·g^−1^, the Equation (6) can be arranged as follows:

(6) dqedt=k2(qe−qt)  2.

where q_e_ (mg·g^−1^) is the adsorption capacity of RhB at equilibrium and q_t_ (mg·g^−1^) is the adsorption capacity of RhB at time t. k_1_ (min^−1^) is the kinetic constant of pseudo-first-order, and k_2_ (g∙mg^−1^∙min^−1^) is the kinetic constant of pseudo-second-order.

Table 4 shows that the adsorption of sample CAP7-27 was consistent with the pseudo-second-order kinetic equation with a linear correlation coefficient R^2^ of 0.99971. The adsorption rate was affected by the square of the number of empty active sites on the surface of sample CAP7–27.

### 3.4. Adsorption Isotherm Studies

**Figure 9:**
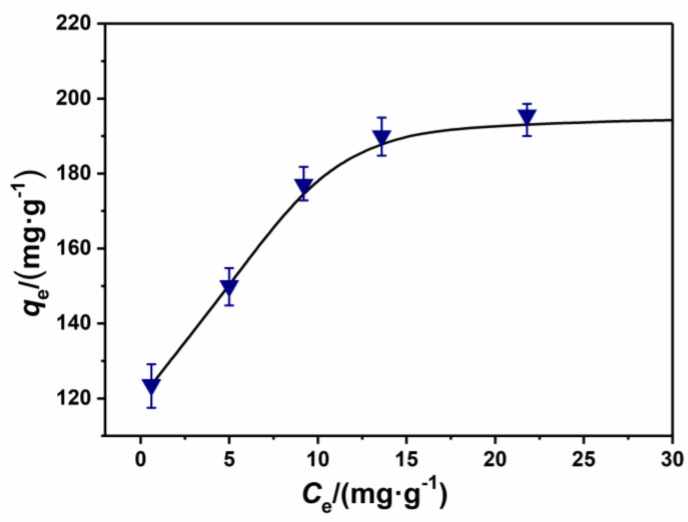
RhB removal capabilities of carbon-covered phosphorus-modified alumina.

Figure 9 indicates that the equilibrium adsorption capacity of CAP7–27 reached 123.6 mg·g^−1^ with an initial RhB concentration of 50 mg·L^−1^. At equilibrium, the concentration of RhB (C_e_) was 0.56 mg·L^−1^, and the RhB removal efficiency reached 98.9%.

The adsorption isotherm data of samples were fitted to the Langmuir isotherm model Equation (7) and the Freundlich isotherm model Equation (8) to study the adsorption mechanism. The two models are generally defined as follows:

Langmuir:

(7) $$q_{e}=\frac{q_{m}K_{L}C_{e}}{1+K_{L}C_{e}},$$

Freundlich:

(8) qe=KFCe1n.

where q_e_ (mg·g^−1^) is the adsorption capacity of RhB at equilibrium, q_m_ (mg·g^−1^) is the maximum adsorption capacity of RhB, C_e_ (mg·L^−1^) is the concentration of RhB at equilibrium, K_L_ is the Langmuir constant, and K_F_ is the Freundlich constant.

Table 5 indicates that the adsorption model of CAP7–27 was consistent with the Langmuir isotherm adsorption model with a linear correlation coefficient R^2^ of 0.9983, indicating that the adsorption of the sample CAP7–27 was a monolayer and homogeneous adsorption process.

## 4. Conclusions

Phosphorus−modified alumina with a pore size of 21.9 nm was synthesized using a coprecipitation method. The carbon-covered, phosphorus-modified alumina CAP7–27 was prepared by impregnating it with glucose and carbonizing it. The specific surface area and pore size of CAP7–27 reached 435.3 m^2^·g^−1^ and 21.2 nm. The RhB adsorption experiment revealed that CAP7–27 had not only an equilibrium adsorption capacity of 198 mg·g^−1^, but also an adsorption rate of 162.5 mg·g^−1^ in 5 min. The kinetics study also showed that the adsorption process was consistent with the pseudo-second-order kinetics and Langmuir isotherm adsorption model. Carbon-Covered phosphorus-modified alumina is a potential highly efficient nanomaterial for the removal of RhB from wastewater.

## Figures and Tables

**Table 1:** Comparison of maximum adsorption capacity for RhB onto various carbon adsorbents.

Adsorbent	Adsorption Capacity (mg·g^−1^)	References
Alumina	3.6	[24]
Carbon-Covered alumina	47.9	[24]
Adsorbent of wheat flour	142.3	[25]
Magnesium silicate/carbon composite	244	[27]
Graphene oxide/silicalite-1 composite	57.0	[28]
Carbon nanotubes	69.0	[29]
Zn/Co ZIFs-derived carbon	116.2	[30]
Fe_3_O_4_/rGO	142.9	[31]
Carbon-Covered phosphorus-modified alumina	198.0	This work

**Table 2:** The data of nitrogen adsorption of phosphorus-modified alumina.

Sample	Specific Surface Area (m²·g^−1^)	Pore Volume (cm^3^·g^−1^)	Most Probable Aperture (nm)
AP0	307.4	0.96	11.8
AP1	356.1	1.17	16.9
AP3	453.6	2.88	20.1
AP5	467.8	3.01	21.2
AP7	496.2	3.03	21.9
AP9	517.9	2.99	21.0

**Table 3:** The data of nitrogen adsorption of carbon-covered phosphorus-modified alumina.

Sample	Specific Surface Area (m²·g^−1^)	Pore Volume (cm^3^·g^−1^)	Most Probable Aperture (nm)
CAP7–23	470.1	2.65	21.3
CAP7–25	440.0	2.46	21.2
CAP7–27	435.3	2.43	21.2
CAP7–30	436.3	2.37	21.3
CAP7–32	441.9	2.34	21.0

**Table 4:** Parameters of kinetics simulation for adsorption on CAP7–27.

Adsorbent	q_e_ (mg·g^−1^)	Pseudo-First-Order Model		Pseudo-Second-Order Model	
		k_1_ (min^−1^)	R^2^	k_2_ (g·mg^−1^·min^−1^)	R^2^
CAP7–27	197.3	0.0075	0.9574	0.0017	0.9997

**Table 5:** Parameters of adsorption isotherms for adsorption on CAP7–27.

Adsorbent	Langmuir Isotherm Model			Freundlich Isotherm Model		
	q_m_ (mg·g^−1^)	K_L_ (L·mg^−1^)	R^2^	K_F_ (mg·g^−1^)(L·mg^−1^)1/n	1/n	R^2^
CAP7–27	195.3	1.28	0.9983	8.298	0.1308	0.9513

## Data Availability

The data presented in this study are available on request from the corresponding author.
